# Predictive Contribution of Clinical Measures of Ankle Neuromuscular Impairments to Ankle Kinematic Deviations During Gait in Children With Cerebral Palsy

**DOI:** 10.1002/jfa2.70195

**Published:** 2026-10-21

**Authors:** Faustyna Manikowska, Sabina Brazevic, Brian Po‐Jung Chen, Marek Jóźwiak, Maria K. Lebiedowska

**Affiliations:** ^1^ Department of Pediatric Orthopedics and Traumatology Gait and Motion Analysis Laboratory Poznań University of Medical Sciences Poznań Poland; ^2^ Motion Analysis Laboratory, Bone and Joint Research Center Chang Gung Memorial Hospital–Linkou Taoyuan City Taiwan; ^3^ Independent Researcher Edmonton Alberta Canada

**Keywords:** ankle, cerebral palsy, gait, hypertonia, kinematics, neuromuscular impairment

## Abstract

**Introductions:**

Children with cerebral palsy (CP) commonly exhibit neuromuscular impairments that affect ankle motion during gait. These include hypertonia, muscle weakness, poor selective motor control, and restricted range‐of‐motion. However, their individual and combined impacts on sagittal ankle kinematics remain unclear. This study aimed to quantify the relative contribution of these impairments on ankle motion during gait in children with spastic CP.

**Methods:**

A total of 132 children with spastic CP, 264 limbs, underwent clinical assessments, and three‐dimensional gait analysis. Clinical tests evaluated muscle strength, hypertonia, selective motor control, and passive ankle range‐of‐motion. Ankle angles were measured at five key gait events: initial contact, loading response, terminal stance, preswing, and mid‐swing. Multiple linear regression models examined the association between impairments and ankle kinematics.

**Results:**

Soleus shortening explained 8% of the variance in ankle position at initial contact. Mid‐swing dorsiflexion was influenced by soleus shortening, triceps surae hypertonia, and impaired plantarflexor selective motor control, accounting for 25% of the variance. The change from initial contact to mid‐swing was similarly explained around 15%, with hypertonia emerging as the strongest predictor. No clinical variables significantly explained stance‐phase kinematics.

**Conclusion:**

Soleus length, plantarflexor hypertonia, and selective motor control impairments partially explain swing‐phase ankle motion but not stance‐phase kinematics. These findings suggest that standard clinical assessments have limited value in predicting weight‐bearing gait function. Comprehensive evaluations incorporating objective biomechanical measures are needed to guide individualized treatment planning in children with CP.

## 1 Intorduction

The ankle joint range of motion (ROM) in the sagittal plane during gait in healthy individuals primarily involves dorsiflexion (upward movement of the foot) and plantarflexion (downward movement of the foot). Muscle activity at the ankle during gait is characterized by distinct phases: plantarflexor muscles are active during the stance phase to provide ankle stability and restrain the forward progression of the tibia. Consequently, dorsiflexion muscles activate during the initial contact and loading response phases, controlling plantarflexion, and again during swing phase to appropriately position the foot [1, 2].

Five critical events were identified during the gait cycle, at which ankle joint positions were calculated (Figure 1). In typically developing individuals, the ankle joint amplitude during the gait cycle is usually around 25°, ranging from approximately 10° of dorsiflexion to 15° of plantarflexion [1, 3]. Typically, ROM consists of three arcs of motion during the stance phase and one during the swing phase. Generally, motion in the stance phase is related to shock absorption and forward body progression, whereas dorsiflexion during the swing phase is crucial for foot clearance. The first rocker or heel rocker, where momentarily the axis rotates around the heel, occurs at the beginning of the stance phase and contributes to shock absorption at initial foot contact. It begins with initial contact (A1 ∼0°) and reaches maximum plantarflexion during the loading response phase (A2 ∼5°). The second rocker or ankle rocker, where momentarily the axis rotates around the ankle, is critical for body progression while the foot is on the ground, reaching maximum dorsiflexion at the end of the terminal stance phase (A3 ∼10°). A sudden change in the direction of foot motion occurs during the pre‐swing phase, ending with maximum plantarflexion (A4 ∼15°). This third rocker or forefoot rocker, where the axis rotates around the forefoot, facilitates toe‐off and accelerates the foot in preparation for swing. Finally, dorsiflexion during the swing phase ensures effective foot clearance, reaching its maximum during mid‐swing (A5 ∼2°).

**FIGURE 1:**
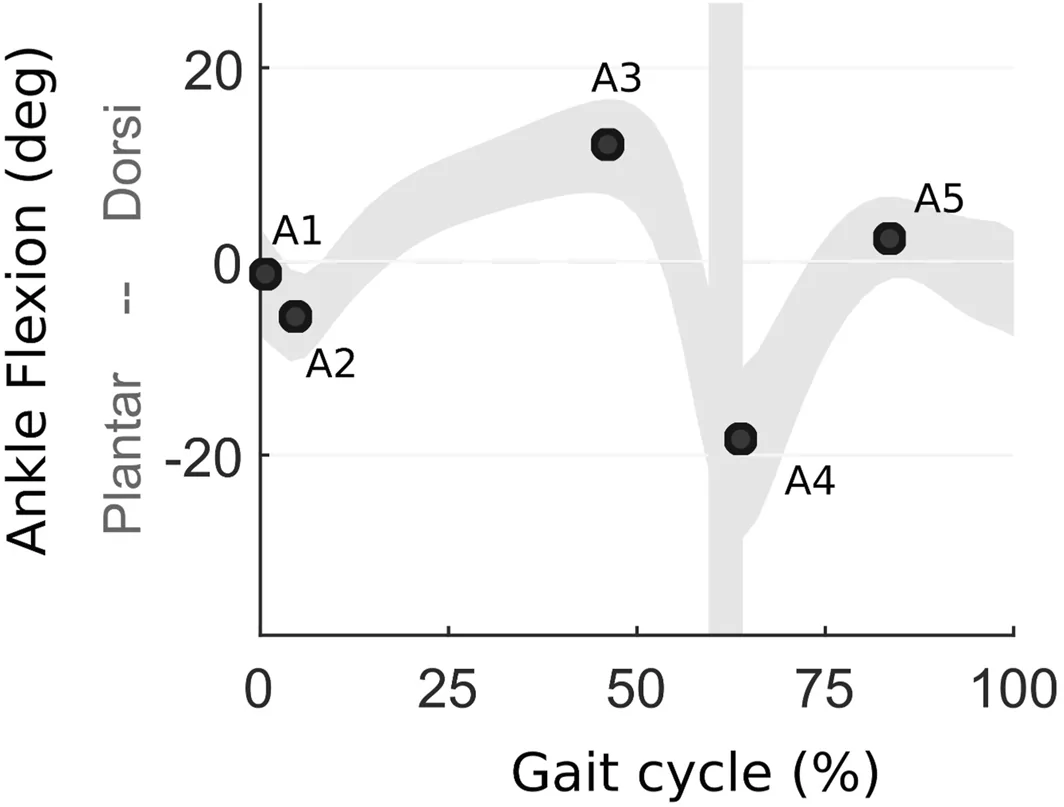
The definitions of ankle positions during the gait cycle were: Initial contact (A1), maximum plantarflexion during loading response (LR) phase (A2), maximum dorsiflexion during terminal stance (TSt) phase (A3), maximum plantarflexion during pre‐swing (PSw) phase (A4), and maximum dorsiflexion during mid‐swing (MSw) phase (A5).

In individuals with cerebral palsy (CP), gait function is frequently impaired as a consequence of brain lesions affecting descending motor control. These neuromuscular impairments may arise from reduced descending neural activation and impaired descending neural inhibition. Reduced neural activation contributes to muscle weakness and may impair skeletal muscle growth relative to skeletal growth, thereby limiting joint range of motion. Impaired descending neural inhibition may increase the sensitivity of the muscle stretch reflex, resulting in spasticity, and may also contribute to impaired selective motor control, with a tendency to move in flexion or extension synergy. These impairments, either individually or in combination, can contribute to muscle shortening and substantially disrupt normal gait. Previous studies have demonstrated that single or combined motor impairments can significantly alter joint kinematics during gait [4, 5, 6, 7, 8].

Common gait deviations at the ankle joint in individuals with CP typically manifest as excessive or restricted plantar or dorsiflexion. Key contributing factors to abnormal gait kinematics include plantarflexor contractures and hypertonia, weakness in plantarflexors or dorsiflexors, pain, proprioceptive deficits, restricted ankle ROM, and persistent knee flexion during stance [1, 9]. Although previous research has established associations between specific impairments and gait abnormalities in CP, most studies have analyzed single impairments independently without detailed examination of their effects on particular gait phases at the ankle joint [8, 10, 11, 12]. Given that multiple impairments commonly coexist in CP, assessments focusing on single impairments may not accurately represent an individual’s functional gait status [13]. Consequently, the precise contribution and relative impact of overlapping impairments, including hypertonia, muscle weakness, restricted ROM, and impaired SMC, on sagittal plane ankle kinematics remain poorly understood. Different impairments may distinctly influence gait kinematics during various phases of the gait cycle. Giving this complexity, identifying the impairment with the greatest influence on gait dysfunction remains challenging and critical for effective clinical intervention.

Human gait reflects the coordinated interaction of multiple joints and muscle groups rather than the isolated function of a single segment. In children with CP, impairments at the hip and knee may substantially influence ankle motion during gait. For example, weak hip extensors, hip flexor contracture, weak knee extensors, knee flexor contracture, and impaired selective motor control at the hip or knee may alter ankle position during stance and swing phases. This multilevel biomechanical interaction is one of the key rationales for comprehensive gait analysis and single‐event multilevel surgery (SEMLS). Nevertheless, selective interventions targeting a single impairment or anatomical level, such as botulinum toxin injection or ankle‐focused surgery, remain common in clinical practice. Therefore, understanding the relationship between clinically measured neuromuscular impairments and ankle kinematic deviations remains clinically relevant, while recognizing that ankle motion may also be influenced by proximal impairments and multilevel gait mechanics.

The present study aimed to systematically evaluate and rank the impact of individual, concurrently existing impairments at the ankle joint on sagittal plane ankle motion during gait. To fulfill this objective, ankle joint positions and ROM through the gait cycle were capture by the instrumented gait analysis, complemented by clinical assessments of muscle strength (MMT), hypertonia (HYP), selective motor control (SMC), and passive knee flexion ROM (CON) in individuals with CP.

## 2 Materials and Methods

### 2.1 Participants

A total of 132 children (age = 11 ± 4 years; 76 males and 56 females) diagnosed as spastic hypertonia CP were enrolled as study participants, and both lower limbs from each participant were analyzed, yielding data from 264 lower limbs. Two lower limbs were excluded from the final analysis because of incomplete ankle kinematic data, leaving 262 lower limbs for analysis. All data collection occurred at a gait and motion analysis laboratory of a local orthopedic and rehabilitation hospital.

### 2.2 Inclusion and Exclusion Criteria

Participants were included if they met the following criteria: (1) no history of botulinum toxin injection or orthopedic surgery within 6 months prior to examination; (2) ability to follow verbal instructions; and (3) independent ambulation. Participants were excluded if they reported pain in the lower limbs or exhibited leg length discrepancy.

Ethical approval was obtained from the Institutional Review Board, and informed written consent was obtained from all participants or their legal guardians prior to enrollment.

### 2.3 Protocol

#### 2.3.1 Clinical Assessments

Clinical examinations included:

— Manual Muscle Testing (MMT): Muscle strength of plantarflexors and dorsiflexors was assessed using the 5‐point grading scale, with scores ranging from 0 (no muscle contraction) to 5 (full strength against maximal external resistance). A muscle strength score below 4 was defined as indicative of impairment [14].
— Muscle Hypertonia (HYP): Muscle tone of plantarflexors and dorsiflexors was assessed with the Modified Ashworth Scale (HYP_AS) and the Tardieu Scale at both the slowest (HYP_V1) and the fastest (HPY_V3) velocities [8, 15]. Scores greater than 0 indicated hypertonia.
— Selective Motor Control (SMC): Evaluated using a 2‐point grading scale, where 2 indicated the ability to perform completely isolated movements, and 0 represented the inability to perform isolated movements. Scores lower than 2 were consider indicative of impairment [16].
— Passive Range of Motion (CON): Passive ROM of plantarflexion and dorsiflexion was measured using a manual goniometer. Deviations from normative angular values considered indicative of impairment [14].

#### 2.3.2 Gait Assessment

An eight‐camera three‐dimensional gait analysis system (six Bonita cameras and two Vero cameras; Vicon Motion Systems Ltd., Oxford, UK) with a sampling rate of 120 Hz was employed. Reflective markers were placed according to the standard Plug‐in‐Gait model. Participants were instructed to walk barefoot along a 10‐m walkway at the self‐selected speed. Kinematic data were collected specifically during preswing (PSw) and initial swing (ISw) phases to measure the ROM and velocity of knee flexion in the sagittal plane during terminal stance (TSt).

### 2.4 Data Analysis

#### 2.4.1 Outcome Measures

The primary outcome measure was ankle motion in the sagittal plane during gait. Five key events were identified within the gait cycle, during which the ankle joint positions were calculated (Figure 1): initial contact (A1), maximum plantarflexion during the loading response (LR) phase (A2), maximum dorsiflexion during the terminal stance (TSt) phase (A3), maximum plantarflexion during the pre‐swing (PSw) phase (A4), and maximum dorsiflexion during the mid‐swing (MSw) phase (A5). In addition, the ranges of ankle motion between these events were analyzed: A1–A2, A3–A4, and A5–A4.

The following impairments, potentially influencing the ankle joint ROM during gait, were identified and analyzed (Table 1):

- Hypertonia of plantarflexors (HYP_TS), assessed using the Ashworth Scale (HYP_AS_TS) and the Tardieu Scale at slow (HYP_TS_v1) and fast (HYP_TS_v3) movement velocities;
- Muscle weakness of plantarflexors (MMT_TS) and dorsiflexors (MMT_DOR);
- Lack of selective motor control of plantarflexors (SMC_TS) and dorsiflexors (SMC_DOR);
- Restricted passive ROM of plantarflexion (CON_TS and CON_SOL) and dorsiflexion (CON_DOR).

**TABLE 1:** The definitions of analyzed impairments.

Impairment	Muscle groups	Outcome measure
Hypertonia (HYP)	Plantarflexors‐triceps surae	HYP_TS_v1
		HYP_TS_v3
		HYP _AS_TS
Lack of selectivity (SMC)	Dorsiflexors	SMC_DOR
	Plantarflexors‐triceps surae	SMC_TS
Muscle weakness (MMT)	Dorsiflexors	MMT_DOR
	Plantarflexors‐triceps surae	MMT_TS
Muscle shortening (CON)	Dorsiflexors	CON_DOR
	Plantarflexors‐triceps surae	CON_TS
	Plantarflexors‐soleus	CON_SOL

### 2.5 Statistical Analysis

Multiple regression analysis, which considers the combined contribution of all identified impairments, was used as proposed by Desloover et al. Therefore, a multiple linear regression model was developed to simultaneously evaluate the impact of selected impairment measures (independent variables) on specific knee ROM (dependent variable). The recommended sample size was estimated using the guideline *n* ≥ 50 + 8*k*, where *k* represents the number of explanatory variables included in the model. Model significance was assessed using analysis of variance (ANOVA and *F*‐test), whereas the significance of individual parameters was evaluated using *t*‐tests. Regression coefficients were calculated using the ordinary lease squares (OLS) method.

Statistical analyses were performed using Statistica 13 (TIBCO Software Inc., Palo Alto, CA, USA), Stata (StataCorp LLC, College Station, TX, USA), and StatXact (Cytel Inc., Waltham, MA, USA), with the significance level set at *p* = 0.05.

## 3 Results

The multiple regression analysis was performed using ankle position and ankle ROM variables as dependent measures to determine the relative contribution of individual impairments in the presence of coexisting multi‐impairments. To improve clinical readability, the dependent variables are presented in the order of the gait cycle: ankle position at initial contact (A1), peak plantarflexion during loading response (A2), peak dorsiflexion at terminal stance (A3), peak plantarflexion during preswing (A4), and peak dorsiflexion during mid‐swing (A5). The overall results of the regression models, categorized according to statistical significance and whether the assumptions of regression analysis were met, are summarized in Table 2. Only statistically significant models that met all assumptions of regression analysis are discussed below.

**TABLE 2:** Summary of multiple regression analysis of effects of selected impairments on increased plantarflexion and dorsiflexion on selected ankle positions (A1–A5) and ROMs (A3–A4, A5–A1, and A2–A1).

Model	Analysis result		
	Statistically significant and model assumptions kept (meet)?	Statistically significant, But assumptions are violated	Statistically NOT significant
Increased plantarflexion			
A1	×		
A2			x
A3		×	
A4		×	
A5	×		
A3–A4			×
A5–A1	×		
A2–A1		×	
Increased dorsiflexion			
A1			×
A2			×
A3			×
A4			×
A5			×
A3–A4		×	
A5–A4			×

*Note:* × showed the category (statistically significant, but assumptions are violated, statistically NOT significant) of the selected positions and ROMs.

For ankle position at initial contact (A1), the regression model was statistically significant and met all regression assumptions (*p* < 0.00001), with 8% of the variance explained by soleus contracture (CON_SOL) (Table 3). Specifically, greater soleus contracture was associated with a more plantarflexed ankle position at initial contact.

**TABLE 3:** Correlation of angle joint rang of motion at A1, A5, and A5–A1.

	*β*	95% CI	*p* value
A1			
A_ROM_DORSI_90	0.21	[0.11; 0.29]	< 0.00001
Adjusted *R* ^2^ = 0.0816, *F* = 21.6178, *p* < 0.00001			
A5			
A_ROM_DORSI_90	0.40	[0.30; 0.50]	< 0.00001
ANKLE_PLANT_V3	−1.33	[−2.42; −0.25]	0.0164
SEL_ANKLE_PLANTAR	−1.52	[−3.03; −0.02]	0.0472
Adjusted *R* ^2^ = 0.2484, *F* = 26.4531, *p* < 0.00001			
A5–A1			
A_ROM_DORSI_90	0.18	[0.12; 0.25]	< 0.00001
ANKLE_PLANT_V3	−1.25	[−2.01; −0.49]	0.0014
Adjusted *R* ^2^ = 0.1537, *F* = 21.9824, *p* < 0.00001			

Abbreviations: *β*, slope of regression, CI, confidence interval at 95%.

No statistically significant regression model meeting all assumptions was identified for peak plantarflexion during loading response (A2), peak dorsiflexion at terminal stance (A3), or peak plantarflexion during pre‐swing (A4) as summarized in Table 2.

For ankle position during swing, represented by peak dorsiflexion during mid‐swing (A5), the regression model was statistically significant and met all regression assumptions (*p* < 0.00001). Three independent predictors, including soleus contracture (CON_SOL), triceps surae hypertonia (HYP_TS_v3), and selective motor control of the triceps surae (SMC_TS), together explained 25% of the variance. Greater plantarflexion during mid‐swing was associated with increased soleus contracture, greater triceps surae hypertonia, and poorer selective motor control. Notably, triceps surae hypertonia and selective motor control were each approximately three times stronger predictors than soleus contracture within this model.

For ankle ROM from initial contact to mid‐swing, represented by the difference between ankle position at mid‐swing and ankle position at initial contact (A5–A1), the regression model was statistically significant and met all regression assumptions (*p* < 0.00001), explaining 15% of the variance. This outcome was significantly influenced by soleus contracture and triceps surae hypertonia, with triceps surae hypertonia emerging as an approximately sixfold stronger predictor than soleus contracture.

Overall, the significant regression models primarily involved ankle position at initial contact and ankle dorsiflexion during swing, whereas no significant models were identified for the stance‐phase events of loading response, terminal stance, or preswing.

## 4 Discussion

The aim of this study was to determine the extent to which individual coexisting impairments on the ankle joint ROM in sagittal plane during gait. We found that increased plantarflexion at initial contact (A1) was associated with decreased soleus muscle length (CON_SOL), this factor accounted for only 8% of the variance (*p* < 0.00001).

Increasing plantarflexion at mid swing (A5) can be explain by a combination of decreased soleus length, triceps surae spasticity, and reduced selectivity of plantarflexors. Together, these impairments explained 25% of the variance, with spasticity and poor selectivity being three times stronger predictors than soleus shortening. Similarly, increased plantarflexion at terminal swing (A5–A1) was related to reduced soleus length and triceps surae spasticity, accounting for 15% of the variance. In this case, spasticity was six times more influential than muscle length. Beyond these findings, no other investigated impairments were identified as significant contributors to changes in ankle dorsiflexion or plantarflexion at the studied gait events. Our results suggest that clinical assessments of neuromotor deficits explain only a portion of ankle joint kinematics abnormalities. Notably, the predictive value of these impairments was confined to the swing phase, when no ground reaction force is present. During this phase, adequate dorsiflexors strength, selective motor control, and absence of plantarflexors hypertonia are critical for achieving foot clearance and appropriate positioning at initial contact [17, 18, 19].

We did not identify significant clinical predictors of ankle pathological kinematics during stance. This may be due to the complex interaction between internal impairments and external forces acting on the limb during weight‐bearing phases. Thus, stance‐phase kinematics likely reflect a combination of neuromotor deficits, joint kinetics, and the individual's adaptive capacity [12, 20]. Previous study has explored the joint influence of muscle morphology and clinical impairment on gait pathology [21]. These findings emphasized that muscle volume, strength, selective motor control, and joint ROM are key contributors to pathological gait kinematics, reinforcing the need for a comprehensive multifactorial approach.

Another possible explanation is that the present analysis was limited to clinical evaluation of impairments around the ankle joint. Clinical examination remains the most commonly used approach in daily practice and continues to guide treatment decision‐making, including focal interventions, such as single‐muscle injection, tendon lengthening, or tendon transfer. However, clinical assessment is not fully quantitative and may be influenced by examiner judgment, ordinal grading, and limited sensitivity, particularly when distinguishing mild impairment or higher levels of muscle strength. Therefore, the lack of significant predictors in stance should not be interpreted as evidence that ankle‐related impairments are unimportant. Rather, it may indicate that conventional clinical measures, when restricted to impairments around a single joint, are insufficient to fully explain dynamic ankle kinematics during gait.

In addition, ankle motion during gait is not determined by ankle impairments alone. Hip and knee kinematics, muscle function, and segmental alignment play important roles in shaping the ankle kinematic pattern through intersegmental coupling and compensatory mechanisms. For example, altered hip or knee flexion during stance may change the functional position of the ankle, influence tibial progression, and affect the demand for ankle dorsiflexion or plantarflexion. Therefore, prediction of ankle gait kinematics based only on clinical examination of ankle‐level impairments, or limited to a single joint or plane, is inherently limited and may represent a potential source of incomplete treatment planning or treatment failure. Our findings further support the importance of comprehensive gait evaluation that integrates clinical examination with quantitative gait analysis and considers interactions among the hip, knee, ankle, and foot.

The role of plantarflexor and dorsiflexor muscle length, tone, strength, and selective motor control have been widely acknowledged as crucial factors influencing gait patterns [22, 23, 24, 25]. However, earlier studies typically evaluated impairments in isolation rather than in combination and without focusing on specific joint positions and ROMs. In contrast, our outcome measures were selected based on the neurophysiological likelihood of impairments contributing significantly to functional gait deficits.

To optimize gait in individuals with CP, individualized treatment strategies should target the preservation of foot rocker function and appropriate ankle joint kinematics and kinetics. Interventions, such as gastrocnemius lengthening and tibialis anterior tendon shortening, have been proposed to improve the ankle ROM during swing [26]. Botulinum toxin injection and various ankle‐foot orthoses have also been shown to enhance [27, 28, 29, 30, 31, 32, 33]. However, reports of overcorrection and insufficient improvement underscore the complexity of treatment planning. Interestingly, interventions not directly targeting the ankle joint, such as selective dorsal rhizotomy or virtual reality training, have also demonstrated positive effects on ankle kinematics [34, 35, 36, 37, 38, 39]. These findings further support the notion that the direct correlation between clinically assessed impairments and functional gait performance remains limited.

### 4.1 Limitations

The primary limitation of this study is that the statistical models focused specifically on ankle joint motion during gait and on neuromuscular impairments of muscles acting primarily around the ankle as identified through standard clinical assessments. Although this ankle‐focused approach allowed us to examine the direct relationship between local impairments and ankle kinematics, ankle motion during gait should not be interpreted as an isolated distal phenomenon. Gait deviations in children with cerebral palsy are multifactorial and result from the combined effects of muscle weakness, muscle shortening or contracture, spasticity, impaired selective motor control, segmental alignment, joint kinetics, and compensatory strategies across multiple lower‐limb segments. Therefore, the limited predictive value observed in the present models may partly reflect the restriction of the analysis to ankle‐level impairments rather than a lack of clinical relevance of ankle impairments themselves.

In particular, impairments at the hip and knee may substantially influence ankle kinematics through intersegmental coupling and compensatory mechanisms. Altered hip or knee flexion during stance may affect tibial progression, the functional position of the ankle, and the demand for ankle dorsiflexion or plantarflexion. Similarly, impaired selective motor control or muscle weakness at the hip and knee may influence limb advancement, foot clearance, and terminal swing ankle positioning. These interactions were not captured in the current model. As a result, the present findings should be interpreted as describing ankle‐focused associations rather than providing a complete explanatory model of ankle kinematics during gait. Therefore, future studies should incorporate a more comprehensive multi‐segmental model, including impairments and kinematics, at the hip, knee, ankle, and foot as well as their interactions during both stance and swing phases.

Another important limitation is the relatively low precision of some clinical measurements used to characterize neuromuscular impairments. Standard clinical assessments, including manual muscle testing, passive range‐of‐motion examination, spasticity grading, and evaluation of selective motor control, are widely used in daily clinical practice and remain important for treatment decision‐making. However, these methods are not fully quantitative and may be influenced by examiner judgment, ordinal grading, limited sensitivity, and ceiling or floor effects. This issue is particularly relevant for manual muscle testing (MMT), especially at higher strength grades. As previously reported by Manikowska et al., differentiation between MMT grades 4 and 5 in children and adolescents with cerebral palsy may be misleading and clinically confusing. To reduce the risk of overinterpreting uncertain distinctions between higher MMT grades, the present study categorized MMT scores of 1, 2, and 3 as indicating muscle strength impairment, whereas scores of 4 and 5 were categorized as indicating no clinically evident impairment. This classification was intended to improve interpretability and reduce potential misclassification related to the limited precision of MMT at higher strength levels. However, by combining grades 4 and 5 into a single nonimpaired category, subtle, or mild weakness may not have been captured, and the predictive contribution of ankle muscle weakness may have been underestimated.

Accordingly, the finding that ankle‐related neuromuscular impairments demonstrated limited predictive value for ankle kinematics during gait should be interpreted with caution. This result does not indicate that ankle impairments are unimportant for gait function. Rather, it suggests that ankle‐related impairments as measured by conventional clinical assessments and analyzed within an ankle‐focused model, explained only part of the variability in ankle kinematics. In clinical practice, focal interventions, such as single‐muscle injection, tendon lengthening, or tendon transfer, are commonly performed based on clinical examination. However, prediction of dynamic gait kinematics based only on clinical evaluation of impairments around a single joint or plane may be limited and could represent a potential source of incomplete treatment planning or unsatisfactory treatment outcomes.

Therefore, our results highlight the limitations of relying solely on conventional clinical tests for treatment selection and support the need for objective assessments to better guide clinical decision‐making. Future research should incorporate more quantitative methods, such as handheld or isokinetic dynamometry, instrumented assessment of spasticity or joint stiffness, dynamic electromyography, advanced three‐dimensional gait analysis, and musculoskeletal modeling. These approaches may improve the precision of impairment measurement and help clarify whether abnormal ankle motion is primarily driven by local ankle impairment, proximal hip or knee dysfunction, altered joint kinetics, or compensatory whole‐limb gait strategies. To assist in identifying potentially meaningful associations, results from models that violated statistical assumptions are included in the appendix for reference (Supporting Information S1: Appendix 1).

## 5 Conclusion

This study investigated the relative contribution of coexisting, clinically evaluated ankle‐related neuromuscular impairments to sagittal‐plane ankle kinematics during gait in children with cerebral palsy. Within this ankle‐focused framework, reduced soleus length, triceps surae spasticity, and poor selective motor control were associated with selected swing‐phase ankle kinematics, including initial contact, mid‐swing, and terminal swing; however, these impairments explained only a modest proportion of the variance, and no significant ankle‐level predictors were identified for stance‐phase kinematics. These findings suggest that standard clinical assessments of ankle‐related impairments have limited explanatory value for predicting weight‐bearing gait function when used in isolation, particularly during stance, where ankle position is also influenced by proximal impairments, whole‐limb compensatory mechanisms, joint kinetics, tibial progression, and weight‐bearing demands. Therefore, ankle kinematics during gait should not be interpreted as the result of ankle impairments alone. A comprehensive, multi‐segmental evaluation approach that includes the hip, knee, ankle, foot, trunk, joint kinetics, and their interactions is needed to better understand pathological gait and support individualized treatment planning in children with cerebral palsy.

## Author Contributions

**Faustyna Manikowska:** conceptualization, data curation, formal analysis, funding acquisition, investigation, methodology, resources, writing – original draft, writing – review and editing. **Sabina Brazevic:** data curation, formal analysis, investigation, project administration, resources, validation, writing – original draft, writing – review and editing. **Brian Po-Jung Chen:** formal analysis, writing – original draft, writing – review and editing. **Marek Jóźwiak:** conceptualization, funding acquisition, project administration, supervision, writing – review and editing. **Maria K. Lebiedowska:** conceptualization, data curation, formal analysis, methodology, supervision, validation, writing – original draft, writing – review and editing.

## Funding

This study was supported by a grant from the Polish National Centre for Research and Development (Narodowe Centrum Badań i Rozwoju [NCBiR]; Grant POIR.04.01.04‐00‐0035/19‐00).

## Ethics Statement

Ethical approval was obtained from the Institutional Review Board, and informed written consent was obtained from all participants or their legal guardians prior to enrollment.

## Conflicts of Interest

The authors declare no conflicts of interest.

## Permission to Reproduce Material From Other Sources

The authors have nothing to report.

## Supporting information


Supporting Information S1

## Data Availability

The data that support the findings of this study are available on request from the corresponding author. The data are not publicly available due to privacy or ethical restrictions.
