# Supplementary material for: Predictive Contribution of Clinical Measures of Ankle Neuromuscular Impairments to Ankle Kinematic Deviations During Gait in Children With Cerebral Palsy
Source: J Foot Ankle Res. 2026 Oct 21;19(4):e70195. doi: 10.1002/jfa2.70195 (PMC13686583; doi:10.1002/jfa2.70195)
Supplement: Supplementary file 1 [file JFA2-19-e70195-s001.docx]

Appendix 1.

Data shown in Table 3 refer to performed analyses, where the estimated values of the coefficients should be approached with great caution since the assumptions of the model are violated (the residuals/errors are not normally distributed).

In this analyses 11% (p < 0.00001) of the variance in ankle angle at terminal stance (A3) can be explained by CON_SOL and HYP_TS_V3. CON_SOL as well as SMC_TS were found to be significant predictive factors in case of A4 (7% of the variance, p < 0.0001) and A2-A1 range (19% of the variance, p = 0.00004).

For A3-A4 of ankle ROM the only variable that showed significant association was MMT_DOR. This parameter can explain 3% of the variance in A3-A4 (p = 0.0073).

In order to explain increasing dorsiflexion analogous analysis was performed (Table 4). In this case just one model showed statistically significant results, however in this case assumptions were violated (similar to data in Table 3). It revealed that 2% (p = 0.0119) of the variance in A3-A4 range can be explained by SMC_TS.

**Table 3. Multiple linear regression analysis with selected ankle positions and ROM as dependent variable (N = 264). INCREASING PLANTARflexion ----- in these models assumptions are violated.**

|  | β | 95% CI | p-value |
| --- | --- | --- | --- |
| **A3** |  |  |  |
| A_ROM_DORSI_90 | 0.25 | [0.15; 0.35] | < 0.00001 |
| ANKLE_PLANT_V3 | -1.20 | [-2.33; -0.06] | 0.0393 |
| Adjusted *R^2^* = 0.1081, *F* = 15.0051, *p* < 0.00001 |  |  |  |
| **A4** |  |  |  |
| A_ROM_DORSI_90 | 0.31 | [0.16; 0.47] | < 0.0001 |
| SEL_ANKLE_PLANTAR | -3.03 | [-5.48; -0.59] | 0.0154 |
| Adjusted *R^2^* = 0.0720, *F* = 9.9585, *p* < 0.0001 |  |  |  |
| **A2-A1** |  |  |  |
| A_ROM_DORSI_90 | -0.08 | [-0.14; -0.03] | 0.0019 |
| SEL_ANKLE_PLANTAR | 1.48 | [0.68; 2.27] | 0.0004 |
| Adjusted *R^2^* = 0.1887, *F* = 11.3514, *p* = 0.00004 |  |  |  |
| **A3-A4** |  |  |  |
| MVC_DORSI | 1.53 | [0.42; 2.65] | 0.0073 |
| Adjusted *R^2^* = 0.0266, *F* = 7.3217, *p* = 0.0073 |  |  |  |

β – slope of regression, CI – confidence interval at 95%

**Table 4. Multiple linear regression analysis with selected ankle positions and ROM as dependent variable (N = 264). INCREASING DORSIflexion – in this model assumptions are violated.**

|  | β | 95% CI | p-value |
| --- | --- | --- | --- |
| **A3-A4** |  |  |  |
| SEL_ANKLE_PLANTAR | 2.17 | [0.48; 3.86] | 0.0119 |
| Adjusted *R^2^* = 0.0230, *F* = 6.4275, *p* = 0.0119 |  |  |  |

β – slope of regression, CI – confidence interval at 95%
